# The development and validation of a conceptual definition of avoidable transitions from long‐term care to the emergency department: A mixed methods study

**DOI:** 10.1002/hsr2.2204

**Published:** 2024-07-04

**Authors:** Greta G. Cummings, Kaitlyn Tate, Jude Spiers, Rowan El‐Bialy, Patrick McLane, Claire Su‐Yeon Park, Tatiana Penconek, Garnet Cummings, Carole A. Robinson, Robert Colin Reid, Carole A. Estabrooks, Brian H. Rowe, Carol Anderson

**Affiliations:** ^1^ Faculty of Nursing, College of Health Sciences University of Alberta Edmonton Alberta Canada; ^2^ Schulich School of Business York University Toronto Ontario Canada; ^3^ Emergency Strategic Clinical Network Alberta Health Services (AHS) Edmonton Alberta Canada; ^4^ School of Nursing University of British Columbia Vancouver British Columbia Canada; ^5^ School of Health and Exercise Sciences University of British Columbia—Okanagan Campus Kelowna British Columbia Canada; ^6^ Department of Emergency Medicine, Faculty of Medicine and Dentistry University of Alberta Edmonton Alberta Canada; ^7^ Alberta Health Services (AHS) Edmonton Alberta Canada

**Keywords:** avoidable transition, long‐term care, patient transfer, transitional care, unnecessary transition

## Abstract

**Background/Objectives:**

Transitions to and from Emergency Departments (EDs) can be detrimental to long‐term care (LTC) residents and burden the healthcare system. While reducing avoidable transfers is imperative, various terms are used interchangeably including inappropriate, preventable, or unnecessary transitions. Our study objectives were to develop a conceptual definition of avoidable LTC‐ED transitions and to verify the level of stakeholder agreement with this definition.

**Methods:**

The *EX*amining *A*ged *C*are *T*ransitions study adopted an exploratory sequential mixed‐method design. The study was conducted in 2015–2016 in 16 LTC facilities, 1 ED, and 1 Emergency Medical Service (EMS) in a major urban center in western Canada. Phase 1 included 80 participants, (healthcare aides, licensed practical nurses, registered nurses, LTC managers, family members of residents, and EMS staff). We conducted semistructured interviews (*n* = 25) and focus groups (*n* = 19). In Phase 2, 327 ED staff, EMS staff, LTC staff, and medical directors responded to a survey based on the qualitative findings.

**Results:**

Avoidable transitions were attributed to limited resources in LTC, insufficient preventive care, and resident or family wishes. The definition generated was: A transition of an LTC resident to the ED is considered avoidable if: (a) Diagnostic testing, medical assessment, and treatment can be accessed in a timely manner by other means; (b) the reasons for a transfer are unclear and the transition would increase the disorientation, pain, or discomfort of a resident, outweighing a clear benefit of a transfer; and (c) the transition is against the wishes expressed by the resident over time, including through informal and undocumented conversations. There was a high level of agreement with the definition across the four participant groups.

**Conclusions and Implications:**

To effectively reduce LTC resident avoidable transitions, stakeholders must share a common definition. Our conceptual definition may significantly contribute to improved care for LTC residents.

## Key points

- Important dimensions in assessing transition avoidability include timely diagnostic testing availability, in‐facility assessment and treatment options, clear transfer purpose, risk/benefit analysis for resident well‐being, and recognition of advanced care goals and informal care preferences.
- Our findings highlighted essential differences between perceptions of unnecessary and avoidable transitions.
- Avoidable transitions were attributed to limited resources in long‐term care, insufficient preventive care, and resident or family wishes.

## 1 INTRODUCTION

Older adults (≥65 years) residing in long‐term care (LTC) facilities often experience changes in health status leading to a transition to an emergency department (ED) for assessment. Residents often receive fragmented care coordination in this setting, and return to LTC in worse condition than when they left.^1^, ^2^, ^3^, ^4^ While certain transitions to the ED are warranted, others can be avoided through appropriate interventions in LTC settings.^5^, ^6^, ^7^ Studies examining LTC‐ED transitions have found that between 18% and 68% of transitions could be classified as avoidable.^6^, ^8^, ^9^, ^10^ Reducing avoidable transfers among this population can mitigate ED crowding and resource use, as well as improve resident health outcomes, and effectiveness and efficiency of the LTC system.^11^, ^12^

Avoidable transitions are variably described as inappropriate, preventable, or unnecessary. They are also defined using certain ambulatory care sensitive clinical conditions, or complex, interacting factors such as resident or family wishes, availability of diagnostic and treatment resources in LTC, clinician availability, and presence of advance directives.^8^, ^9^, ^13^, ^14^, ^15^ “Potentially avoidable hospitalizations” has been used to classify inappropriate transfers based on clinical conditions including pneumonia, urinary tract infection, congestive heart failure, chronic obstructive pulmonary disease or asthma, dehydration, and pressure sores that could be managed through evidence‐based care in LTC.^6^, ^15^, ^16^, ^17^ Ethnographic work by Vassy suggests that individual providers differ substantially in their understandings of what constitutes a legitimate case for ED care,^18^ while broader literature shows providers' judgments of the degree to which a patient belongs in ED impacts care and compassion for that patient.^19^, ^20^ Similar observations have been made among paramedics.^21^, ^22^ Clear and substantiated definitions of avoidable LTC‐ED transitions will support researchers, decision‐makers, and healthcare practitioners (HCPs) to provide optimal care and reduce unnecessary use of limited healthcare resources. Above all, clarifying transition terminology is essential to develop viable, sustainable interventions in this area.

Understanding how all individuals involved in LTC‐ED decision‐making (residents, family members, and HCPs) define avoidable transitions is critically important. Yet, limited research has examined the definition of avoidable transitions from the perspectives of stakeholders involved in decision‐making that leads to LTC‐ED transitions. In this mixed‐methods study, we aimed to understand the LTC decision‐making processes related to whether, or not, to send a resident to ED for acute care. Our objectives were to describe the development of a conceptual definition of avoidable resident LTC‐ED transitions using qualitative data from LTC nurses (licensed practical nurses's [LPN] and registered nurse's [RN]), healthcare aides, and family members, and validate this definition using quantitative survey data from physicians, paramedics, and nurses working in LTC, Emergency Medical Services (EMS), and ED.

## 2 METHODS

### 2.1 Research design

The findings presented here are part of a mixed‐methods study funded by the Covenant Health and their Network of Excellence in Seniors’ Health and Wellness. The *EX*amining *A*ged *C*are *T*ransitions (EXACT) study used an *exploratory sequential mixed‐methods design*, which consists of qualitative analysis of data followed by a development phase in which the researcher develops a tool or approach, such as a survey instrument, that is then tested quantitatively.^23^

We employed qualitative methods in Phase 1 to explore perceptions of avoidable LTC‐ED transitions, definitions of necessary and avoidable transitions, and their differences, and the complex decision‐making processes leading up to them. A conceptual definition of avoidable transfers was developed based on qualitative findings. In Phase 2, we used descriptive survey results for validation of the definition and further exploration of associated concepts identified in Phase 1, such as what may contribute to necessary and avoidable transitions. We obtained the University of Alberta Health Research Ethics Board (Pro 00051101) and operational approvals before data collection.

### 2.2 Phase 1: Qualitative exploration

Phase 1 ran from February through August 2015. We employed methods guided by principles of focused ethnography to explore perceptions of avoidable transitions from LTC to the ED.^24^, ^25^, ^26^ This method allows for the exploration of a specific topic across geographic locations, while prioritizing two kinds of data collection (namely interviews and focus groups).^24^, ^25^, ^26^

#### 2.2.1 Setting

We examined the characteristics of 26 LTC facilities within a major city in western Canada, which were included in a previous study conducted by members of our research team.^27^, ^28^ We selected six facilities for maximum variation in transfer rates and number of beds. Three large‐size facilities (>200 beds) and three small‐size facilities (<200 beds) were included. Three facilities were publicly owned and operated, one facility was private nonprofit and two were private for‐profit facilities.

#### 2.2.2 Sample

The sample included 80 participants in 25 interviews and 19 focus groups, consisting of 20 healthcare aides, 14 LPNs, 21 RNs, 10 LTC managers, 6 family members of residents, and 9 EMS members. Participants must have worked in the facility or service for at least 1 year, and had experience with one potentially avoidable transfer, or be a family member of a resident transferred to a local ED in the past year. Older adults residing in LTC were considered as potential participants; however, the comparatively high level of cognitive impairments in this population, the unexpected nature of emergency transitions, and the limited resources available to conduct timely interviews made qualitative interviewing of individuals residing in LTC impractical. Participant recruitment occurred through posters, in‐person presentations by the research team, and snowball sampling. Family members of residents who experienced an ED transition were recruited via invitations to LTC Family Councils. Written informed consent was obtained from all participants. Persons who could not communicate in English were excluded.

#### 2.2.3 Data collection and analysis

Concurrent data collection and analysis allowed emerging findings to guide further data generation. The semistructured interview/focus group guide can be viewed in Appendix A. A moderator (J. S. or R. E.) and an observer (research coordinator or trainee) conducted the focus groups and individual interviews. Field notes were taken during the interviews and focus groups. Interviewers also debriefed after the interviews and focus groups. The duration of the interviews ranged from 40 to 90 min, while the focus group discussions were approximately 1–2 h. Interviews were digitally recorded, transcribed verbatim, and deidentified. We used NVIVO‐10® to manage data analysis.

Analysis followed principles of inductive constant comparison.^29^ Three team members (R. E., K. T., J. S.] independently completed initial open and NVIVO coding, and categorization. We examined categories to generate themes and scrutinized focus group observational notes to identify areas of hidden disagreement between participants. Regular team meetings were held to discuss and reach an analytic consensus. When we reached analytic saturation and no new information emerged during data collection,^25^ we used the remaining two focus groups for verification of our findings. Research strategies of theoretical sampling, concurrent data generation and analysis, theoretical sensitivity, and the use of multiple data sources helped incrementally build and ensure rigor.^30^ Emerging qualitative findings were discussed with the research advisory panel, consisting of LTC and ED physicians, nurse practitioners, clinical nurse specialists and educators, EMS managers, and researchers. The definition of avoidable transitions was a synthesis of findings and multiple discussions among analysts, research team, and advisory panel until the team agreed that the definition was clear and resident‐centered.

We followed the Consolidated Criteria for Reporting Qualitative Studies Checklist (see File S1).

### 2.3 Phase 2: Confirming perspectives through surveys with stakeholders

The quantitative phase ran from September 2015 through August 2016 and included a survey developed to assess the validity and generalizability of Phase 1 findings. The survey was pretested with two classes of graduate and undergraduate nursing students to improve readability and clarity.

#### 2.3.1 Sample and setting

We obtained operational approval before conducting the survey in 16 contracted LTC facilities, with Edmonton Zone EMS, Edmonton Zone Facility Living Medical Directors, and 1 acute care ED. We used convenience sampling to recruit participants.

#### 2.3.2 Measures

The survey comprised 46 items and 6 constructed vignettes. In this paper, we report results from three items and six vignettes within two domains (1) level of agreement with our conceptual definition of avoidable LTC‐ED transitions using a Likert‐type scale from 1 (*strongly disagree*) to 6 (*strongly agree*) and (2) perceptions of avoidability or necessity of transitions using vignettes. For each constructed vignette, participants were asked if each transition was necessary and if it could have been avoided. See Appendix B for survey details.

#### 2.3.3 Survey distribution

To ensure the anonymity of participants, survey links were provided to LTC managers and EMS supervisors to distribute via mass staff mailing lists. We adapted Dillman et al.'s email strategy to invite participants to complete the survey using three email reminders following the invitation.^31^

#### 2.3.4 Analysis

Data were analyzed using IBM SPSS Statistics 26 (SPSS Inc.). Continuous data are reported as means and standard deviations (*M* (SD)) when normally distributed, and median and interquartile ranges when not normally distributed. Categorical or dichotomous data are reported as percentages (%). Responses to survey questions were compared by participant group (ED, EMS, LTC, MD). Differences among group means for agreement with the conceptual definition of avoidable transitions were tested using (nonparametric) Kruskal–Wallis tests, considering that (a) all data per group per each definition were not normally distributed and (b) sample sizes among groups differed. Where differences were significant, Dunn–Bonferroni post hoc analysis was performed to determine which specific group means differ.

We used the Checklist for Reporting of Survey Studies, which can be found in File S2.

## 3 RESULTS

### 3.1 Phase 1—Qualitative results

Three overarching themes pertaining to perceptions of avoidable transitions emerged from our qualitative data: *limited resources in LTC*; *insufficient preventive care*; and *resident and family wishes.* Across all three themes, differences between participants' perceptions of an unnecessary or avoidable transition emerged. See Table 1 for supporting quotes for each theme.

**Table 1:** Supporting quotes for qualitative themes.

Theme	Supporting quote
Limited diagnosis and treatment options	It's all about the speed in which things happen. And that's why most times we have to send them out, because we don't have the capacity to get things done quick. And if it's somebody who's deteriorating rapidly, we have nothing that we can do for them. We can't do IVs, which would help boost up their blood pressure. We only have clysis, which you can only run so fast. So, it's better to send them out and not take a chance of them deteriorating to the point where we would have to send them anyways. B‐LPN1
	Probably because families want to know the reason why this is happening to the parent. **Probably the resources in the facility are not enough**. Or probably they want answers right away. “Why is my mom like this?” I do understand in long‐term care, you cannot diagnose right away what's happening to them, because we don't have those right‐away resources, like for x‐rays, blood work, and all that. We can't even do IV here. Long‐term care is not allowed. We can only give IMs, sub‐cutaneous fluid, and POs. Or probably they don't want their family to die that soon. If they could keep their family alive, then they'll do that. E‐RN1
Insufficient preventive care	Okay, so there's this one lady,—like she hadn't been draining that morning. And I came back the next day, and she still had like low or no output. And I was like “well, how come no one changed—I was questioning evenings, right? the evening shift—how come they didn't change it? What did they do? They pushed some fluids—there was still no output. And they didn't change the catheter. So, I came the next day and I changed the catheter. And she did start draining but she was so lethargic, almost unresponsive, so we had to send her to the hospital. D‐LPN1
	You are looking at the charting, you are looking at his intake and output and everything seems dandy—like he should not be dehydrated if he's having one hundred percent of his meals and he's having over a liter of ah, fluids a day, right? Ah, then there is something—then there is a question. Ah, did he really have that much, or what's going on? … or was it just kind of—you know grossly calculated so it wasn't really accurate…, so there are those type of things that I think could be avoided altogether. F‐LPN1
Resident and family wishes	I think a lot of the times, the families are the ones that don't really see the light and they say that they have to go, whereas sometimes I [think] that it's not really necessary, but the family are saying they need to go. A‐RN2
	Even though [a transfer] might be unnecessary in terms of medical treatment, that no intervention might be needed, that trip to the hospital still does answer the question of, is there something more here? Is there something that can be dealt with? So, in that sense, it is necessary. Family Member
	This perception of a necessary transition contrasted many HCPs in our study, as evidenced by the following quote:
	…Based on their goals of care, [we're] not going to treat it, or not going to continue, so why do we need to send them and find that information out anyway … sometimes I think that diagnostics don't necessarily need to happen. A‐MAN1

Abbreviations: HCP, healthcare practitioner; IM, intramuscular; IV, intravenous; LPN, licensed practical nurse; PO, oral; RN, registered nurse.

#### 3.1.1 Limited diagnosis and treatment options in LTC

Facilities varied in their provision of onsite diagnostic testing such as mobile X‐ray. Sometimes LTC residents were transferred to the ED to obtain a diagnosis. Limited treatment options in LTC also supported participants' perceptions that transferring residents is sometimes the only option, even when required treatment is within the LTC‐based LPN and RN's scope of practice.

#### 3.1.2 Insufficient preventive care

Participants highlighted how delayed identification of change in condition (e.g., hydration) along with the use of insufficient or suboptimal preventive interventions in LTC, could result in avoidable ED transitions.

#### 3.1.3 Resident and family wishes

Participants strongly perceived that family requests frequently resulted in avoidable transitions. Family perceptions of avoidability did not always align with those of HCPs, or with previous informal and even formal conversations with residents and HCPs about care preferences. HCPs often felt that clinical assessment and Goals of Care Designation orders should determine the clinical focus of care and treatment. Family members sometimes felt that diagnostic testing warranted a transfer, while staff reported that the need for transfer was determined by the potential for treatment.

## 4 AVOIDABLE DEFINITION DEVELOPMENT RESULTS

Conceptually, our qualitative data suggested a difference between participants' notions of avoidability and necessity when describing LTC‐ED transitions. A transfer that is medically necessary at time of decision to transfer might have been avoided by earlier, preventive measures. As such, a transfer could be both necessary and avoidable.

The following definition, including three parts, was generated by research team members based on multiple discussions about the themes, as described above, identified in qualitative interviews.

A transition of a LTC resident to the ED is considered avoidable if:

1. Diagnostic testing, medical assessment, and treatment can be accessed in a timely manner by other means (e.g., interfacility transfer, mobile X‐ray, community paramedicine programs, etc.).
2. The reasons for a transfer are unclear and the transition would increase the disorientation, pain, or discomfort of a resident, outweighing the clear benefit of a transfer.
3. The transition is against the wishes expressed by the resident over time, including through informal and undocumented conversations.

### 4.1 Phase 2—Quantitative results

Data from 327 surveys were used for quantitative data analyses. After deleting 252 cases with monotone missing values in which the survey was only accessed but not responded to at all, we ended up with a working sample of 327 usable survey responses.

#### 4.1.1 Demographics

Respondents included 40 ED members (RNs, physicians), 130 EMS personnel (EMTs, paramedics, operations supervisors), 134 LTC staff (LPNs, RNs, managers, administrators), and 23 LTC medical directors (MD; physicians). More female participants (*n* = 194, 59.3%) responded than male participants (*n* = 120, 36.7%). Most participants ranged in age from 30s (*n* = 99, 30.3%), 40s (*n* = 88, 26.9%) to 50s (*n* = 54, 16.5%). See Table 2 for Phase 2 participant characteristics. The total number of valid values excludes missing values per demographic information.

**Table 2:** Phase 2 participant characteristics (*N* = 327).

			Groups				
Characteristics *n* (%)^a^/*M*(SD)			ED	EMS	LTC	MD (LTC)	Total
Profession	LPN		0 (0.0)	n/a	34 (25.6)	0 (0.0)	34 (10.5)
	RN		24 (60.0)	1 (0.8)	87 (65.4)	0 (0.0)	112 (34.7)
	Physician		16 (40.0)	n/a	7 (5.3)	23 (100.0)	46 (14.2)
	Paramedic		n/a	76 (59.8)	n/a	n/a	76 (23.5)
	EMT		n/a	46 (36.2)	n/a	n/a	46 (14.2)
	EMR		n/a	0 (0.0)	n/a	n/a	0 (0.0)
	Supervisor		n/a	4 (3.1)	n/a	n/a	4 (1.2)
	Other		0 (0.0)	n/a	5 (3.8)	0 (0.0)	5 (1.5)
	ECCURT		n/a	13 (10.2)	n/a	n/a	13 (10.2)
Shift^b^	Day		27 (67.5)	112 (86.2)	87 (64.9)	0 (0.0)	226 (69.1)
	Evening		19 (47.5)	46 (35.4)	48 (35.8)	0 (0.0)	113 (34.6)
	Night		16 (40.0)	72 (55.4)	15 (11.2)	0 (0.0)	103 (31.5)
Role	Clinical		32 (80.0)	n/a	86 (65.2)	20 (87.0)	138 (70.8)
	Administrative		0 (0.0)	n/a	13 (9.8)	0 (0.0)	13 (6.7)
	Both		0 (0.0)	n/a	33 (25.0)	3 (13.0)	44 (22.6)
Management position	Yes		3 (7.7)	n/a	47 (35.3)	3 (13.0)	53 (27.2)
Gerontology education	Completed		3 (7.5)	n/a	25 (19.1)	2 (8.7)	30 (15.5)
Working years [median (IQR)]			9.000 (12.00)	10.00 (12.00)	10.00 (11.00)	20.00 (20.00)	10.00 (13.00)
Working period at this facility	≥6 months		n/a	n/a	127 (96.2)	22 (100.0)	149 (96.8)
Education	Certificate		0 (0.0)	30 (23.6)	8 (6.0)	1 (4.3)	39 (12.1)
	Diploma		2 (5.1)	73 (57.5)	50 (37.6)	0 (0.0)	125 (38.8)
	Bachelors		20 (51.3)	21 (16.5)	57 (42.9)	0 (0.0)	98 (30.4)
	Masters		5 (12.8)	3 (2.4)	10 (7.5)	2 (8.7)	20 (6.2)
	Doctoral		12 (30.8)	0 (0.0)	8 (6.0)	20 (87.0)	40 (12.4)
Gender	Male		16 (40.0)	72 (55.4)	14 (10.4)	18 (78.3)	120 (36.7)
	Female		23 (57.5)	51 (39.2)	115 (85.8)	5 (21.7)	194 (59.3)
	Transgender		0 (0.0)	0 (0.0)	0 (0.0)	0 (0.0)	0 (0.0)
	Other		0 (0.0)	0 (0.0)	0 (0.0)	0 (0.0)	0 (0.0)
	Prefer not to disclose		0 (0.0)	3 (2.3)	4 (3.0)	0 (0.0)	7 (2.1)
Age	20–29		11 (27.5)	31 (24.4)	5 (3.8)	0 (0.0)	47 (14.6)
	30–39		10 (25.0)	56 (44.1)	31 (23.3)	2 (8.7)	99 (30.7)
	40–49		12 (30.0)	27 (21.3)	43 (32.3)	6 (26.1)	88 (27.2)
	50–59		5 (12.5)	11 (8.7)	32 (24.1)	6 (26.1)	54 (16.7)
	60–69		2 (5.0)	2 (1.6)	19 (14.3)	7 (30.4)	30 (9.3)
	≥70		0 (0.0)	0 (0.0)	3 (2.3)	2 (8.7)	5 (1.5)
Subtotal			40 (12.2)	130 (39.8)	134 (41.0)	23 (7.0)	327 (100.0)

Abbreviations: ECCURT, emergency medical services continuing care urgent response team; ED, emergency department; EMR, emergency medical responder; EMS, Emergency Medical Services; EMT, emergency medical technician; IQR, interquartile range; LPN, licensed practical nurses; LTC, long‐term care; MD, medical directors; n/a, not applicable; RN, registered nurses.

^a^ Percentages were calculated after deleting missing values per each variable.

^b^ Multiple responses were allowed.

#### 4.1.2 Agreement with the definition of avoidable transitions across groups

The four participant groups each strongly agreed with the overall conceptual definition, *M*(SD) of 5.3 (1.0) (Likert‐type scale from 1 = *strongly disagree* to 6 = *strongly agree*). LTC staff participants had significantly lower agreement with the conceptual definition than EMS and ED staff, respectively (*p* < 0.001). Differences in agreement between other groups were nonsignificant (Appendix C).

**Figure 1:**
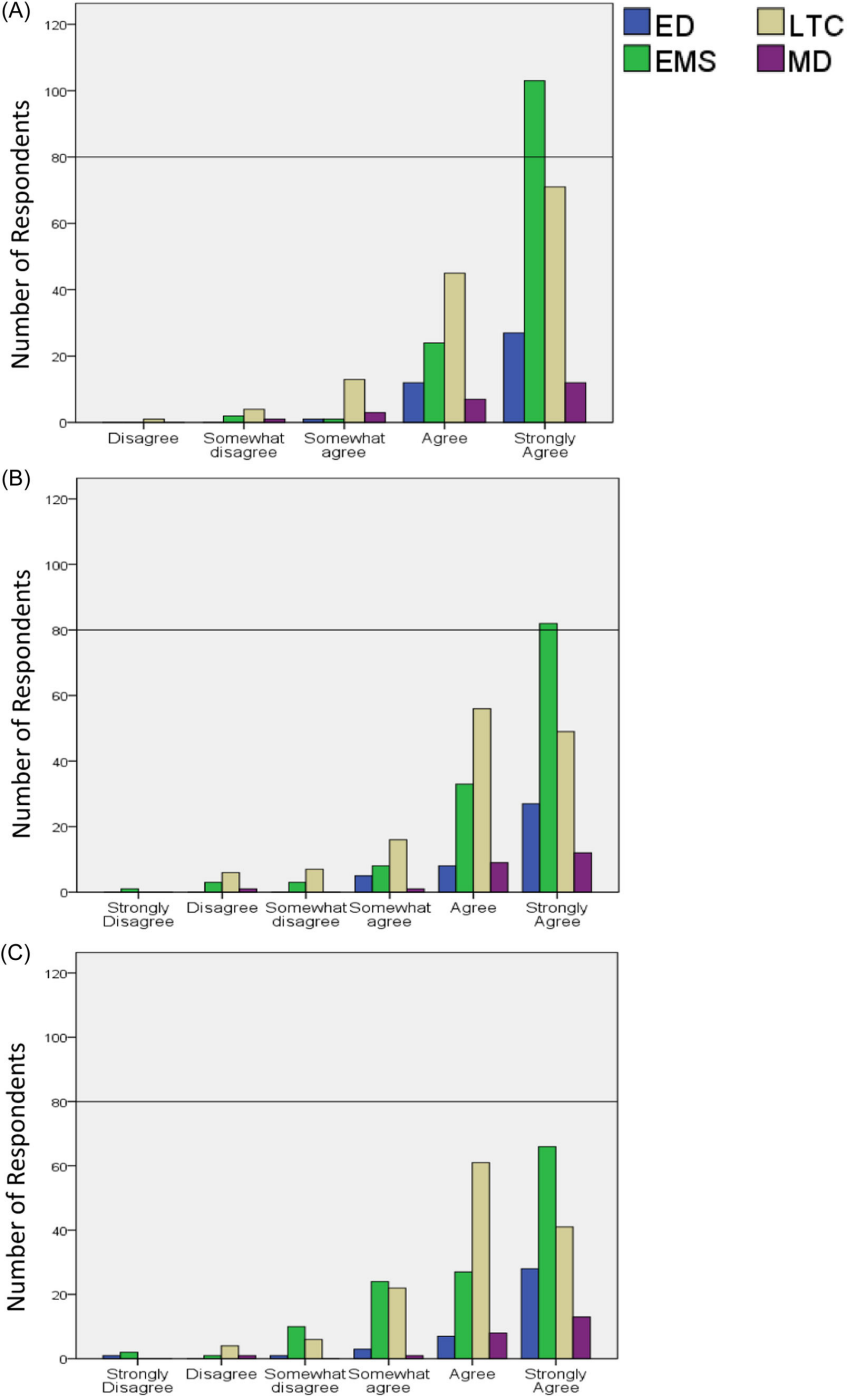
Agreement levels with the three components of The Older Persons' Transitions in Care study's definition of avoidable transitions across groups. (A) Diagnostic testing, medical assessment, and treatment can be accessed in the LTC facility in a timely manner by other means (e.g., portable X‐ray, community paramedics, nurse practitioners, etc.). (B) The reasons for a transfer are unclear and if the transfer would increase the disorientation, pain, or discomfort of a resident, outweighing any benefit of a transfer. (C) It is against the wishes expressed by the resident over time, including through informal and undocumented conversations. ED, emergency department; EMS, Emergency Medical Services; LTC, long‐term care; MD, medical directors.

Differences in agreement per definition component among groups were statistically significant, *p* < 0.001. Although overall agreement levels were high, for definition part A (*diagnostic testing, medical assessment, and treatment can be accessed in a timely manner by other means* [*e.g., interfacility transfer, mobile X‐ray, community paramedicine programs, etc.*]), LTC and MD showed statistically lower agreement than EMS, respectively (*p* < 0.05). For definition part B (*the reasons for a transfer are unclear and the transition would increase the disorientation, pain, or discomfort of a resident, outweighing a clear benefit of a transfer*), LTC showed statistically lower agreement than EMS and MD, respectively (*p* < 0.05). For definition, part C (*the transition is against the wishes expressed by the resident over time, including through informal and undocumented conversations*), LTC showed statistically lower agreement than ED (*p* < 0.001). A comparative illustration per definition component per group is presented in Figure 1.

#### 4.1.3 Perceptions of avoidability and necessity of transition situations across groups

**Figure 2:**
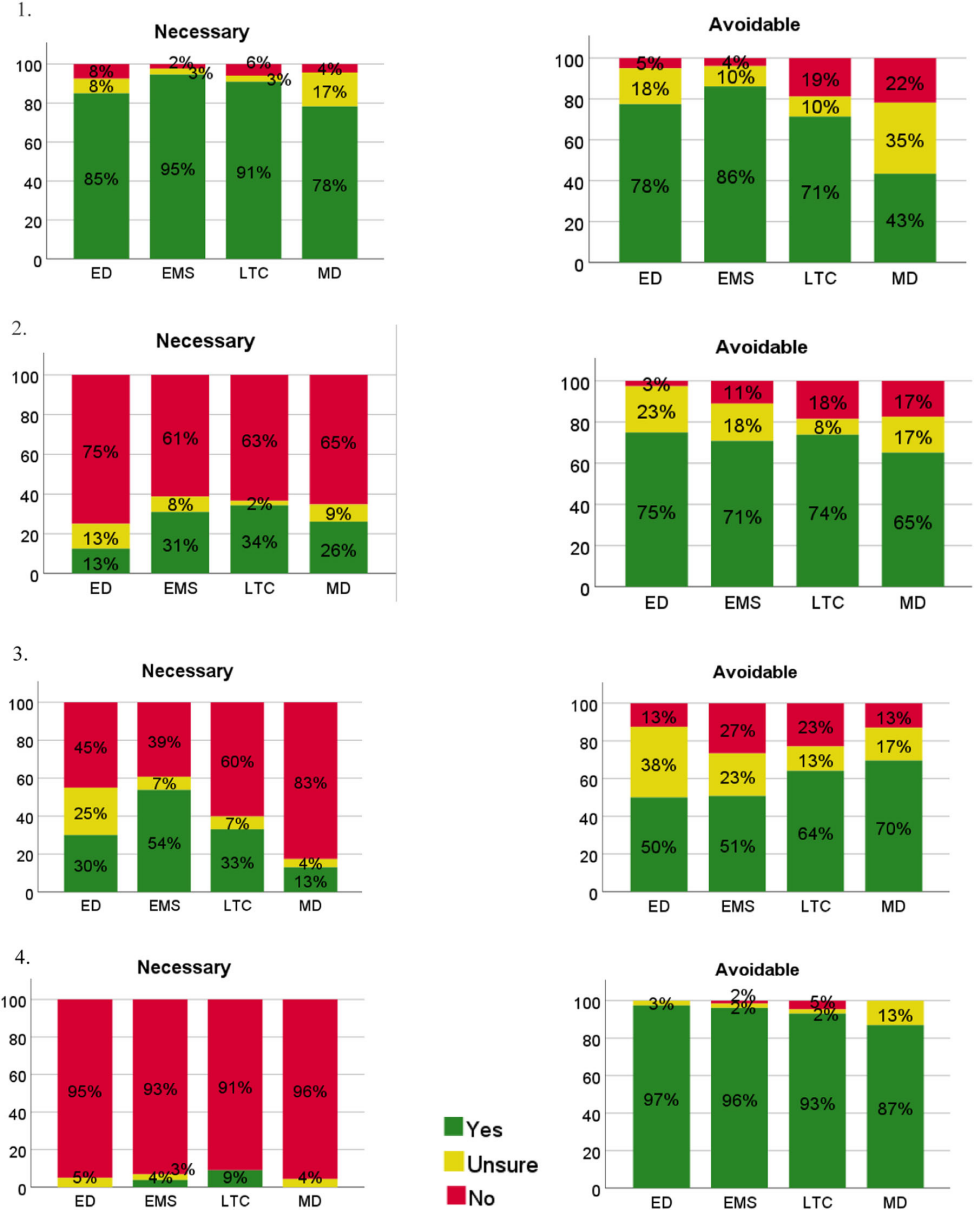
Participants' perceptions of avoidability and necessity in LTC to ED transitions. 1. A resident's condition began to change, but the signs were not recognized or reported promptly. The residents condition continues to deteriorate significantly. Treatment options available in the LTC cannot address the condition. 2. A resident's Goals of Care Designation is Comfort level (C1) and their condition has changed without a clear cause. Family caregivers request a transfer for diagnostic reasons. 3. A resident develops malaise, general weakness, and is not eating or mobilizing as normal. Clinical assessment shows no clear cause for the symptoms. 4. A resident's family is not regularly involved in the resident's care and they have not observed the gradual changes in a resident's condition. When the family visits the resident, they are alarmed by the change in the resident's state compared to their last visit and request that the resident be transferred to the ED. ED, emergency department; EMS, Emergency Medical Services; LTC, long‐term care; MD, medical directors.

Notable differences between participants' perceptions of avoidability and necessity were evident across transition situations. For example, responses for the situation, “A resident develops malaise, general weakness, and is not eating or mobilizing as normal. Clinical assessment shows no clear cause for the symptoms,” were mixed, generating the most “unsure” responses for whether the transition was avoidable (*n* = 65, 20.2%). Approximately half of participants agreed that this transition was unnecessary (*n* = 168, 51.5%). Participant perceptions of whether certain transition situation vignettes were avoidable or unnecessary can be viewed in Figure 2 and Table 3.

**Table 3:** Participant perceptions across groups of whether a transition situation from LTC to ED was necessary or avoidable (*N* = 327).

Situations	Group	Transfer *n* (%)					
		Necessary			Avoidable		
		Yes	No	Unsure	Yes	No	Unsure
A resident's condition began to change, but the signs were not recognized or reported promptly. The residents condition continues to deteriorate significantly. Treatment options available in the LTC cannot address the condition.	Total	296 (90.8)	15 (4.6)	15 (4.6)	248 (76.1)	37 (11.3)	41 (12.6)
	ED	34 (85)	3 (7.5)	3 (7.5)	31 (77.5)	2 (5)	7 (17.5)
	EMS	123 (94.6)	3 (2.3)	4 (3.1)	112 (86.2)	5 (3.8)	13 (10)
	LTC	121 (90.2)	8 (6.0)	4 (3.0)	95 (70.9)	25 (18.7)	13 (9.7)
	MD	18 (78.3)	1 (4.3)	4 (17.4)	10 (43.5)	5 (21.7)	8 (34.8)
A resident is dying and no treatment available can change this. Family members have difficulty accepting the resident's death and request a transfer to the ED.	Total	29 (8.9)	294 (89.9)	4 (1.2)	298 (92.0)	11 (3.4)	15 (4.6)
	ED	2 (5.0)	38 (95)	0 (0.0)	39 (97.5)	0 (0.0)	1 (2.5)
	EMS	2 (1.5)	125 (96.2)	3 (2.3)	120 (92.3)	1 (0.7)	7 (5.4)
	LTC	23 (17.2)	110 (82)	1 (0.7)	119 (88.8)	9 (6.7)	5 (3.7)
	MD	2 (8.7)	21 (91.3)	0 (0.0)	20 (87)	1 (4.3)	2 (8.7)
A residents Goals of Care Designation is Comfort level (C1) and their condition has changed without a clear cause. Family caregivers request a transfer for diagnostic reasons.	Total	97 (29.8)	209 (64.1)	20 (6.1)	231 (72.2)	43 (13.4)	46 (14.4)
	ED	5 (12.5)	30 (75)	5 (12.5)	30 (75)	1 (2.5)	9 (22.5)
	EMS	40 (30.8)	79 (60.8)	10 (7.7)	90 (69.2)	14 (10.8)	23 (17.7)
	LTC	46 (34.3)	85 (63.4)	3 (2.2)	96 (71.6)	24 (17.9)	10 (7.5)
	MD	6 (26.1)	15 (65.2)	2 (8.7)	15 (65.2)	4 (17.4)	4 (17.4)
A resident develops malaise, general weakness, and is not eating or mobilizing as normal. Clinical assessment shows no clear cause for the symptoms.	Total	129 (39.6)	168 (51.5)	29 (8.9)	185 (57.5)	72 (22.4)	65 (20.2)
	ED	12 (30)	18 (45)	10 (25)	20 (50)	5 (12.5)	15 (37.5)
	EMS	70 (53.8)	51 (39.2)	9 (6.9)	65 (50)	34 (26.2)	29 (72.5)
	LTC	44 (32.8)	80 (59.7)	9 (6.7)	84 (62.7)	30 (22.3)	17 (12.7)
	MD	3 (13)	19 (82.6)	1 (4.3)	16 (69.6)	3 (13)	4 (17.4)
A resident's falls, has no sign of injury, and the Goals of Care Designation is Resuscitation (R1).	Total	18 (5.5)	300 (92.3)	7 (2.2)	278 (87.4)	23 (7.2)	17 (5.3)
	ED	2 (5.0)	34 (85)	4 (10)	33 (82.5)	1 (2.5)	6 (15)
	EMS	9 (6.9)	118 (90.8)	3 (2.3)	107 (82.3)	11 (8.5)	9 (6.9)
	LTC	6 (4.5)	126 (94)	0 (0.0)	116 (86.6)	10 (7.5)	2 (1.5)
	MD	1 (4.3)	22 (95.7)	0 (0.0)	22 (95.7)	1 (4.3)	0 (0.0)
A resident's family is not regularly involved in the resident's care and they have not observed the gradual changes in a resident's condition. When the family visits the resident, they are alarmed by the change in the resident's state compared to their last visit and request that the resident be transferred to the ED.	Total	17 (5.2)	302 (92.6)	7 (2.1)	302 (94.4)	8 (2.5)	10 (3.1)
	ED	0 (0.0)	38 (95)	2 (5)	38 (95)	0 (0.0)	1 (2.5)
	EMS	5 (3.8)	121 (93.1)	4 (3.1)	123 (94.6)	2 (1.5)	3 (2.3)
	LTC	12 (9.0)	121 (90.2)	0 (0.0)	121 (90.2)	6 (4.5)	3 (2.2)
	MD	0 (0.0)	22 (95.7)	1 (4.3)	20 (87)	0 (0.0)	3 (13)

*Note*: Mssing values per each variable have been deleted before analyses.

Abbreviations: ED, emergency department; EMS, Emergency Medical Services; LTC, long‐term care; MD, medical directors.

## 5 DISCUSSION

The EXACTs study is the first mixed‐methods study where a conceptual definition of avoidable LTC‐ED transitions was developed, validated and *mutually agreed upon* among stakeholders from involved care settings. Our definition goes beyond those based purely on clinical conditions.^32^, ^33^ Key dimensions for assessing the avoidability of a transition include availability of timely diagnostic testing, potential assessment and treatment within the facility, clarity of purpose for the transfer, a risk/benefit analysis of resident wellbeing, and recognition of advanced care goals of care and informal care wishes. Critically, our findings highlighted essential differences between perceptions of unnecessary and avoidable transitions.

Although agreement with our definition was high across all participant groups, LTC staff had significantly lower agreement than other stakeholders. This highlights that a transition *from* LTC for LTC staff may be seen as unavoidable when resources are not available onsite to effectively care for a resident, but the transition *to* the ED may be seen as avoidable to ED personnel when care could be provided outside of the ED. Other reports demonstrate differences in HCP perspectives on the need for ED transition and issues in transitional care based on care setting.^34^ Our qualitative findings demonstrated that family members perceived necessity differently when reporting that diagnostics was a sufficient reason to transition, regardless of treatment options or intent. It is vital to reconcile discrepant views to provide care that is needed, ethical, consistent across services, and congruent with previously expressed wishes of the individual when they cannot easily advocate for themselves due to a combination of cognitive impairments, communication challenges, and acute health changes. Using our definition as common ground among care providers and across care settings may improve communication and collaboration among care team members and informal caregivers, in an effort to support safe and efficient transitions in practice and desired health outcomes.^35^ Communication issues can arise from cultural differences across care environments, the presence of multiple rotating medical teams in hospitals, and frequent turnover of nursing and administrative staff in LTC, which, in turn, negatively affect resident care.^36^ Investing in interdisciplinary and cross‐setting approaches to prevent LTC‐ED transitions is warranted.^37^, ^38^ Research is needed to examine how these interventions provide the necessary structure, norms and communication pathways to relay more consistent resident information and to improve relationships, and reconcile differing expectations around transitional care for older adults.^27^, ^39^, ^40^ Crucially, even when applying clear definitions of avoidable ED transitions, healthcare providers must be well‐resourced to ensure that judgments of a transition as avoidable after it occurs do not result in poor treatment of the patient (e.g., inattention, lack of compassion).

Our research findings highlight commonalities and distinctions between unnecessary and avoidable transitions. A transition can be both unnecessary and avoidable, or necessary at the time of transition decision, but perceived as avoidable had effective preventive care been provided earlier. Distinguishing unnecessary and avoidable transitions can support identification of cases where residents require early action in preventive care or, for example, when improved communication and counseling for family members around resident prognosis may be required. Transition situations identified as necessary but avoidable typically related to the need for early preventive care. RN and/or NP outreach programs whereby LTC residents receive regular, proactive visits, and regular reviews of their charts and care plans, have been well‐received by LTC staff and demonstrated a reduction in transitions to the ED.^37^

Transitions occurring due to family requests were often perceived as unnecessary by HCPs. Better documentation around advance care planning and early, ongoing conversations among the entire LTC team are warranted. However, existing reviews on interventions to reduce or improve LTC‐ED transitions highlight the lack of interventional research aimed at improving LTC staff/family relations, and family understandings of resident health status, despite other research reporting these tensions as a significant issue in transitional care for older adults.^37^, ^41^ A state‐of‐the‐art review identified that family engagement models to proactively educate informal caregivers about care transitions that older adults with dementia can experience, contain these key components—evaluate goals of care against the care continuum, improve communication across and within care settings, and involve informal caregivers in a collaborative team environment.^42^ These recommendations should be adopted and evaluated for more successful LTC‐ED transitions.

The transition situation generating the most mixed and “don't know” responses described a resident experiencing general malaise and ambiguous symptom presentation. The need for improved geriatric education and training in various healthcare settings is longstanding, especially pertaining to atypical or ambiguous symptom presentation among older adults.^27^, ^43^, ^44^ Short‐form geriatric assessments could be implemented in LTC to support thorough assessments during acute or unclear changes in resident health.^27^, ^45^ Cross‐setting educational sessions may be warranted to improve HCPs' geriatric assessment skills and confidence, as well as relationships among providers. In addition, training for ED providers and EMS personnel (likely to judge scenarios as avoidable in our data) on the realities of LTC could also help ED and EMS understand LTC decisions to transfer, recognize that they do not always have access to all relevant information, and decrease the likelihood that a transition may be prematurely judged avoidable. Our findings that LTC, EMS, and ED have differing understandings of whether an ED transition is avoidable when presented with the same scenario, show that differing understandings across services, and not just information gaps, impact coordination of care. Knowing and addressing this is essential for health systems to provide holistic consistent care across services and for services to interact well.

An agreed‐upon definition of avoidable transitions provides a strong basis to develop and test quality measures to better identify, evaluate, and reduce avoidable transitions in practice.^38^ Specifically, our definition can be used to develop structural and process indicators relevant to the intersection of healthcare services and professions, such as availability and timeliness of diagnostic testing, clinical assessment, and *quality and clarity* rather than just the existence of documentation around the reason for transition and advanced care planning.^38^, ^46^

### 5.1 Strengths and limitations

Our study findings have an inherent limitation of generalizability due to purposeful sampling for qualitative exploration, convenience sampling for the quantitative survey, and data collection restricted to a certain region of the world. However, study participants generally reflected the workforce in LTC facilities.^47^, ^48^ The definition proposed in this study was developed rigorously based on stakeholder perspectives and may function as an informed shared decision‐making rationale among all LTC HCPs, to develop and validate interventions to effectively reduce avoidable transfers between LTC and ED. Second, the study was conducted in Canada, where healthcare services are delivered without charge to the individual patient. Results may differ in other jurisdictions. The research was performed before the COVID‐19 pandemic when advance directives were prioritized and unnecessary and avoidable transfers were strongly discouraged. Validation of the current finding in this new “era” may be warranted. A major strength of our study is the inclusion of quantitative and qualitative data from all care providers involved in LTC to ED transitions.

## 6 CONCLUSION AND IMPLICATIONS

Clarifying the concepts and terminology used to describe transitions is essential to develop viable, sustainable interventions to effectively reduce avoidable transitions from LTC to ED and improve coordination of care across services. We rigorously developed and validated a definition of avoidable LTC‐ED transitions, which may support shared decision‐making among all HCPs involved in transitions in care. Further, we provide a critical distinction between unnecessary and avoidable LTC‐ED transitions. This new knowledge may significantly contribute to advancing LTC science for future intervention studies and evidence‐informed policy‐making in transitional care for older adults.

## AUTHOR CONTRIBUTIONS

The study's concept and design were contributed by Jude Spiers, Greta G. Cummings, Garnet Cummings, Brian H. Rowe, Robert Colin Reid, Carole A. Robinson, Carol Anderson, and Carole A. Estabrooks. Carol Anderson, Kaitlyn Tate, Rowan El‐Bialy, and Jude Spiers were responsible for data acquisition. Jude Spiers, Greta G. Cummings, Garnet Cummings, Brian H. Rowe, Robert Colin Reid, Carol Anderson, Carole A. Estabrooks, Patrick McLane, Rowan El‐Bialy, Kaitlyn Tate, and Claire Su‐Yeon Park analyzed and interpreted the data. Kaitlyn Tate, Jude Spiers, Greta G. Cummings, and Tatiana Penconek drafted the manuscript, and all authors contributed to its critical revision for important intellectual content.

## CONFLICT OF INTEREST STATEMENT

The authors declare no conflict of interest.

## ETHICS STATEMENT

University of Alberta Health Research Ethics Board (Pro 00051101) and operational approvals were obtained before data collection. Written informed consent was obtained from all participants.

## TRANSPARENCY STATEMENT

The lead author Greta G. Cummings affirms that this manuscript is an honest, accurate, and transparent account of the study being reported; that no important aspects of the study have been omitted; and that any discrepancies from the study as planned (and, if relevant, registered) have been explained.

## Supporting information

Supporting information.

Supporting information.

## Data Availability

Data and materials can be requested from the corresponding author.

## APPENDIX A FOCUS GROUP/INTERVIEW GUIDES

**Each session for nurses, HCAs, families will begin with a brief overview of the OPTIC (The Older Persons’ Transitions in Care) findings to set the context of the discussion and the focus on avoidable transfers to ED**

### Overview of OPTIC Study findings

The OPTIC study examined characteristics of successful transitions between long term care facilities (LTC) and the Emergency Department (ED) by tracking residents who were transferred and asking the LTC nurses, managers, EMS staff, and ED staff to complete surveys about the resident and transfer. Over 637 resident transfers were tracked.

Most transfers were necessary, but 160 transfers were identified as potentially avoidable by at least one health care provider. However there was little agreement across healthcare providers as to what it means for a transfer to be “avoidable”. So, while the vast majority of transfers ARE necessary, we want to try to understand the decision‐making process that leads to the decision‐to‐transfer, and the factors that might influence a decision to pursue or avoid a transfer. We want to try to clarify what a necessary or an avoidable transfer means.

Many decisions‐to‐transfer are clear. For example, an 88 year‐old female resident falls and fractures her hip. She is in pain and LTCs clearly do not do surgery. A decision‐not‐to‐transfer can also be clear. For example, a 90 year‐old resident who is dying with his family present does not wish to be moved to hospital and is receiving appropriate supportive palliative care in the LTC. However, other transfer decisions are more ambiguous. Through this study we will explore the factors involved in decision‐making in ambiguous cases. We want to develop a definition of necessary and avoidable transfers and a guideline to help in making decisions.

We will be talking to RNs/LPNs as the clinical authority, HCAs as they often are the first to notice a change in condition, the family councils, the NH medical directors group and EMS. Our ultimate goal is to determine areas that we can find ways to support decision‐making to minimize the number of avoidable transfers. In this focus group/interview, we are going to ask you to talk about the things that influenced your decision‐making in ambiguous cases.

**The following questions in the interview protocols are guides only and may be used in any order, according to the flow of the discussion. Some groups respond well to a concrete case based “think aloud” process (questions 1 and 2 and not the other questions) while others will prefer a more generalized discussion around the various domains of decision‐making factors. Questions will be selected according to situational need.**

### RN/LPN Nursing Staff

**Introduction:** *following informed consent procedures*:

- Provide overview of OPTIC
- Rowan: Reminder “I am not a nurse, so I might ask you to explain more for me”

1. *Demographics: Year qualified as a RN/LPN; Diploma/BScN; Years in Facility; Years in LTC; Age; Country of Original education as a Nurse. How did you choose a career here in LTC? Years in UNIT and FACILITY*
2. In general, how do you tell the **difference between a clear need to transfer and a situation when it's not clear whether a transfer is necessary?** *Probe for critical factors at individual, family, institutional and professional domains.*
3. Can you think of a **transfer** that **you were not sure about**? Describe the resident scenario. PROBE
  a. How was Change in condition detected, by whom, time line and ease in transferring information?
  b. How do you **decide whether a S&S is problematic or a normal or expected variation**: how is this documented? *How do you weigh all of these factors?*
  c. P**oints** were there where you might have had choices about transferring?
  d. What made you decide one way or another?
  e. What was difficult about this decision‐making process: *Family, Goals of Care, and Resources.*
  f. Retrospect being 100% effective, what might have been done to prevent this transfer? If not avoidable, why not?
  g. Advantages/disadvantages of NOT transferring, i.e., keeping, a resident in ambiguous situations? *Probe for the advantage to whom, short‐ and long‐term benefits*
4. Can you tell me about a situation when you **THOUGHT** you would transfer but eventually **decided not to transfer**? Tell me about the event starting from who noticed a change in condition. PROBE:
  a. Resident status, goals of care, family involvement and reaction, clinical assessment, team work among nursing team and with physician.
  b. What happened with the resident/family/Dr that made you decide one way or another?
  c. What was your main concern when you were trying to decide? Why?
5. **Individual nurse factors** (skill, competence and perceptions):
  a. What factors do you take into consideration when you determine whether a resident's health status warrants a transfer?
  b. What criteria is there to determine if a Symptom, condition change or abnormality is problematic or an acceptable variation? E.g., check medication regimen for potential adverse drug reactions, looking at resident history
  c. Use of **clinical pathway guidelines**
  d. Extent of consultation with other RN/LPN
  e. What knowledge or skills would help you/colleagues better recognize, assess and manage changing conditions?
  f. Where are there points at which a decision‐to‐transfer could be avoidable?
6. **Care philosophy and Goals of Care**:
  a. What are the main driving forces that determine need to transfer: When Goals of Care (GoC) are M1, how much flexibility is there to vary this? *If GoC are completed in acute care, how often is the second page of the form detailing the discussion leading to the decision completed with adequate information?*
  b. What policies, guiding principles, or expectations exist here about keeping residents rather sending to ED? *Probe for perspectives around palliative care/dying.*
  c. **What are your perceptions of the appropriateness of caring for residents at the end of life here?**
  d. **How does your assessment of the resident's quality of life or even projected life expectancy influence your decision‐making?**
  e. Do you tend to try to influence families/resident decisions to transfer? How: probe for anticipatory preparation of declining condition, reassurance of interventions to keep comfortable, explication of likely actions and waiting time and outcome in ER.
  f. What is the difference between the goals of care and the old DNR/no code system? Given that the DNR was a legal document and the goals of care are medical orders, has this change affected your practice? Do goals of care give you more room to make your own judgement about whether a person should be transferred or not?
7. **Families**
  a. Do you see any trends in the families/decision makers of residents who are adamant about need to transfer in ambiguous situations? *Probe for*: why no differences or closeness/location, understanding of residents’ clinical condition, readiness/denial/guilt and pressure from other family members
  b. How do you try to work with them throughout the resident's stay and during a change of condition to prepare them for what is happening, and to ensure the best decision about transfer?
  c. How do you balance advocating for the best interests of the residents and the families wishes in situations of ambiguous transfers?
  d. Tell me about a time when a family member disagreed with your assessment of a resident. How did you react?
8. **Physician/EMS Services and ER department**
  a. What kind of response do you get from EMS/ER in ambiguous transfer situations? Why? *Probe* for ageism, understanding of LTC resources, inter‐professional respect, EMS asking Nurse to call physician. Probe for how the nurse defends her assessment and decision.
  b. What kind of feedback do you get from acute care about appropriateness of transfer? What would be helpful?
  c. Does availability of NP make any difference?
  d. How does the relationship you have with the physicians influence transfer decisions?
9. **Resources**
  a. What resources are there regarding what to do when a resident becomes ill or worsens?
  b. What are your choices/options in sending residents via ambulance versus planned dispatch for services such as CT after a fall etc. Probe for availability, whether it is considered, rush to transfer.
  c. At what point do you think that the nursing home environment cannot meet the needs of the resident? Why?
10. **Work environment factors:**
  a. What considerations are important in terms of the work environment in your decision‐making?
    i. E.g., workload (both yours and HCA), competing needs from other residents, ability to delegate work when resident's condition changes.
  b. What benefits are there in deciding to transfer or NOT to transfer a resident?
  c. Can you talk about the culture of **collaboration** for nurses – extent to which they refer/use each other for **second opinions?**
  d. **How does the team work environment in the unit (LPN/HCA) affect decisions to transfer?**
11. What would it take to avoid a transition to hospital? Why?
12. What is one realistic change that could be made in this facility that might reduce the number of avoidable transfers?
13. Are there any other comments group members would like to make before we end?
14. Closing: Summary and brief overview of ideas presented: Appreciation and offering token of gratitude in recognition of their time and effort ($25 gift certificate).

### Healthcare Aides

**Introduction**: *following informed consent procedures;* introduction of group moderators, study purpose and intent of the focus group discussions. Remind participants to keep any information confidential after leaving the group. Provide overview of OPTIC results.

The following questions are guides only and may be used in any order, according to the flow of the discussion as per protocol for LPN/RN protocol.

1. *Demographics: Years in Facility; Years in LTC; Age; If an Immigrant to Canada, ask if qualified as LPN/RN in country of origin; Age*
2. Could you either think about the last resident you looked after who was transferred to ED or resident transferal in general. Tell me what happened. Probe for assessment, communication, resident factors, work environment.
3. Can you walk me through what usually happens before a decision to transfer is made?
4. Whose role is it to observe, assess, document and report information about resident condition change? How well does this work – thinking of transfer situations. Probe: what kind of assessments do you do? Skin integrity, vital signs?
5. How does the communication between you and the nurse influence the decision‐making process? What would help this? Probe: How do you document any changes you notice in the resident? Written or verbal communication?
6. What characteristics of the LPN or RN do you see as contributing to good decisions? *Probe for clinical skills in assessment, confidence, teams work, values, panic or wait and see attitude.*
7. How does your knowledge of the resident influence the decision‐making process? Why or why not?
8. You are in critical role in monitoring residents and noticing changes. What/how do nurses/managers advise you in terms of what to look for and when to report observations to a nurse or what changes to anticipate? How does the RN/LPN use your knowledge of the resident when making a decision to transfer?
9. Do you feel comfortable telling the RN/LPN whether you think a resident should stay at the facility or be transferred?
10. How does the family fit into this decision‐making process?
11. How does the work environment influence these decisions: staffing levels, workload, cooperation/delegation and resources such as radiology/lab tests?
12. Is there any benefit in NOT transferring a resident? What benefits are there in transferring a resident?
13. What do you think is the difference between a necessary transfer and an avoidable transfer?
14. What changes would it be possible to make to reduce the number of avoidable transfers?

Closing: Summary and brief overview of ideas presented: Appreciation and offering token of gratitude in recognition of their time and effort ($25 gift certificate).

### Managers/Directors

**Introduction:** informed consent procedures, introduction of interviewer, study purpose and intent of the discussion. Provide overview of OPTIC results.

The following questions are guides only and may be used in any order, according to the flow of the discussion.

1. *Demographics so we can describe the sample: Years as manager in Facility; Years in Facility; Years in LTC; Disciplinary background (RN, Occ Health, PT etc.). Highest educational level obtained. Age; If immigrant, country of origin ‐?nursing education*
2. **Define**/differentiate between necessary and potentially avoidable transfers?
3. **Assess the necessity** of the transfer in NH?
4. Track transfers to ED in your facility?
5. What proportion is avoidable? What are the financial implications of avoidable transfers – who pays? Who decides and on what grounds is a transfer necessary?
  a. How does the funding model for this facility (public/private/small/large) influence decisions to transfer?
6. Think about the **characteristics and practices of really effective nurse DM** around avoidable transfers. Think of a time you saw one of your nurses do a very effective transfer assessment in an ambiguous situation.
  a. General circumstances leading up to this incident?
  b. What did this nurse do that was so helpful at the time?
  c. Why was this so helpful in making a determination?
  d. How experienced is the nurse?
7. Think of a time when **a nurse, in your opinion, shown ineffective clinic DM** – the sort of action which if repeated, would indicate that the nurse was not effective at assessing the need to transfer?
8. What points in the decision‐making process are critical in decisions to transfer or keep residents?
9. What factors influence the decision at these critical points?
10. Can you talk about the culture of collaboration for nurses – extent to which they refer/use each other for second opinions?
11. Use of clinical pathway guidelines – UTI, falls, pneumonia
12. Interprofessional issues: interactions with EMS/ER: Probe for attitudes, knowledge of EMS/ER about LTC and capabilities, ageism,
13. **Feedback process** regarding the **appropriateness** of transfers for your nurses in your facility
14. **Solutions** are you trying to implement to **address these challenges to** effective decision‐making around transfers?
15. What feasible changes would you think are necessary to minimize avoidable transfers?
  a. Nurse factors: skills, competence, knowledge,
  b. System factors: policies, protocols
  c. Staffing factors
  d. Resources/equipment factors: e.g. lab/radiology/accessibility of Dr./NP
16. What one feasible change would make the most impact on minimizing avoidable transfers?

Closing: Summary: Appreciation & Gift certificate

### Family Council

**Introduction:** informed consent procedures, introduction of interviewers, study purpose and intent of the discussion. Provide overview of OPTIC results.

The following questions are guides only and may be used in any order, according to the flow of the discussion.

1. How are you involved in decisions to transfer to ED?
2. To what extent are residents and families able to articulate their goals of care and preferences before anything happens?

a. How often is this reviewed? Is it adhered to?

3. Thinking of a time when your family member was transferred to the ED: What did the staff identify as the reason for transfer? Was this early or late in the changing condition of your family member?
4. What was the nature of the communication and consultation you have with the LTC staff to come to a decision about transferring?
  a. When were you contacted?
  b. Were your or your family member's preferences taken into account?
5. Do you have experience of a transfer decision for your family member that could have been avoided? How? At what points in the process could this decision have been avoided? *Probe for timeliness, assessment/management/inter‐professional communication.*
6. What feasible changes could minimize avoidable transfers?

Closing: Summary and brief overview of ideas presented: Appreciation and offering token of gratitude in recognition of their time and effort ($25 gift certificate).

### Paramedics

1. How do you define necessary and avoidable transfers?
2. What do you think contributes to avoidable transfers? *Probe nurse, resident, family, resource, or work environment factors.*
3. What happens when you are summoned to an LTC and you feel that it is not necessary to transfer the resident to the ED?
4. What trends have you observed over the past 3 years in terms of number and nature of potential avoidable transfers?
5. What feasible changes could minimize avoidable transfers?

### Medical Directors Group

Introduction; Informed consent procedures, overview of OPTIC findings, summary of themes of qualitative analysis of data from nurses, HCA and family interviews.

1. What are your thoughts on themes in this analysis?
2. Do you track transfers in your facility?
3. Do you assess whether particular transfers are necessary or avoidable?
4. Do you have a sense of what proportion are avoidable?
5. What trends have you observed over the past 3 years?
6. Other than the ideas we presented, at what points do decision‐making processes lead to avoidable transfers?
7. What nurse, resident, family, resource, or work environment factors are contributing to this?
8. What feasible changes could minimize avoidable transfers?

Closing: Summary and brief overview of ideas presented: Appreciation and offering token of gratitude in recognition of their time and effort ($25 gift certificate).

### Emergency Medicine Services (EMS) Group

**Introduction:** informed consent procedures, introduction of interviewer, study purpose and intent of the discussion. Provide overview of OPTIC results.

The following questions are guides only and may be used in any order, according to the flow of the discussion.

1. Response to analysis
2. How do you define necessary and avoidable transfers?
3. What trends have you observed over the past 3 years in terms of number and nature of potential avoidable transfers?
4. Other than the ideas we presented, at what points do decision‐making processes lead to avoidable transfers?
5. What nurse, resident, family, resource, or work environment factors contribute to this?
6. What feasible changes could minimize avoidable transfers?

Closing: Summary and brief overview of ideas presented: Appreciation and offering token of gratitude in recognition of their time and effort ($25 gift certificate).

## APPENDIX C

See Table C1.

**Table C1:** Agreement level with the definitions of avoidable transitions across groups (*N* = 327).

Definitions	Level of agreement	Groups *n* (%)				Total *n* (%)
		ED	EMS	LTC	MD	
(a) Diagnostic testing, medical assessment, and treatment can be accessed in the LTC facility in a timely manner by other means (e.g., portable X‐ray, community paramedics, nurse practitioners, etc.).	*Strongly agree*	27 (12.7)	103 (48.4)	71 (33.3)	12 (5.6)	213 (65.1)
	*Agree*	12 (13.6)	24 (27.3)	45 (51.1)	7 (8.0)	88 (26.9)
	*Somewhat agree*	1 (5.6)	1 (5.6)	13 (72.2)	3 (16.7)	18 (5.5)
	*Somewhat disagree*	0 (0)	2 (28.6)	4 (57.1)	1 (14.3)	7 (2.1)
	*Disagree*	0 (0)	0 (0)	1 (100)	0 (0)	1 (0.3)
	*Strongly disagree*	0 (0)	0 (0)	0 (0)	0 (0)	0 (0)
	*M*(SD)	5.7 (0.5)	5.8 (0.5)	5.4 (0.8)	5.3 (0.9)	*H*(3) = 24.484**
(b) The reasons for a transfer are unclear and if the transfer would increase the disorientation, pain, or discomfort of a resident, outweighing any benefit of a transfer.	*Strongly agree*	27 (15.7)	82 (48.2)	49 (28.8)	12 (7.1)	170 (52.0)
	*Agree*	8 (7.5)	33 (31.1)	56 (52.8)	9 (8.5)	106 (32.4)
	*Somewhat agree*	5 (16.7)	8 (26.7)	16 (53.3)	1 (3.3)	30 (9.2)
	Somewhat disagree	0 (0)	3 (30.0)	7 (70.0)	0 (0)	10 (3.1)
	Disagree	0 (0)	3 (30.0)	6 (60.0)	1 (10.0)	10 (3.1)
	*Strongly disagree*	0 (0)	1 (100)	0 (0)	0 (0)	1 (0.3)
	*M*(SD)	5.6 (0.7)	5.4 (1.0)	5.0 (1.1)	5.3 (0.9)	*H*(3) = 21.834**
(c) It is against the wishes expressed by the resident over time, including through informal and undocumented conversations.	*Strongly agree*	28 (18.9)	66 (44.6)	41 (27.7)	13 (8.8)	148 (45.3)
	*Agree*	7 (6.8)	27 (26.2)	61 (59.2)	8 (7.8)	103 (31.5)
	*Somewhat agree*	3 (6.0)	24 (48.0)	22 (44.0)	1 (2.0)	50 (15.3)
	*Somewhat disagree*	1 (5.9)	10 (58.8)	6 (35.3)	0 (0.0)	17 (5.2)
	*Disagree*	0 (0.0)	1 (16.7)	4 (66.7)	1 (16.7)	6 (1.8)
	*Strongly disagree*	1 (33.3)	2 (66.7)	0 (0.0)	0 (0.0)	3 (0.9)
	*M* (SD)	5.5 (1.0)	5.1 (1.1)	5.0 (1.0)	5.4 (0.9)	*H*(3) = 16.444**
Total	*n* (%)	40 (12.2)	130 (39.8)	134 (41.0)	23 (7.0)	327 (100.0)
	*M* (SD)	5.6 (0.8)	5.4 (1.0)	5.1 (1.0)	5.3 (0.9)	*H*(3) = 20.585**

Abbreviations: ED, emergency department; EMS, Emergency Medical Services; LTC, long‐term care; MD, medical directors.

** *p* < 0.01.
