# Supplementary material for: The development and validation of a conceptual definition of avoidable transitions from long‐term care to the emergency department: A mixed methods study
Source: Health Sci Rep. 2024 Jul 4;7(7):e2204. doi: 10.1002/hsr2.2204 (PMC11224026; doi:10.1002/hsr2.2204)
Supplement: Supplementary file 1 — Supporting information. [file HSR2-7-e2204-s002.docx]

**Supplementary File 1 The Consolidated Criteria for Reporting Qualitative Studies (COREQ) Checklist**

|  | **Guide questions/description** | **Remarks** | **Page no.** |
| --- | --- | --- | --- |
| Domain 1: Research team and reflexivity | | | |
| *a*). *Personal Characteristics* | | | |
| 1. Interviewer/ facilitator | Which author/s conducted the interview or focus group? | A moderator (JS, RE) and an observer (research coordinator or trainee) conducted the focus groups and individual interviews. | 9 |
| 2. Credentials | What were the researcher’s credentials? *E.g*. PhD, MD | The researchers’ credentials are as follows:  GGC: PhD  KT: PhD  JS: PhD  RE: MSc  PM: PhD  CSP: MsN  TP: MN  GC: MSc  CAR: PhD  CR: PhD  CAE: PhD  BHR: MD  CA: BScN | 1 |
| 3. Occupation | What was their occupation at the time of the study? | The researchers’ occupations are as follows:  GGC: Academic, Dean, Professor  KT: Academic  JS: Academic  RE: PhD candidate  PM: Senior researcher  CSP: Graduate student  TP: PhD candidate  GC: Academic  CAR: Academic  CR: Academic  CAE: Academic  BHR: Academic  CA: Chief Zone Officer at Alberta Health Services | 1 |
| 4. Gender | Was the researcher male or female? | The researchers’ gender are as follows:  GGC: Female  KT: Female  JS: Female  RE: Female  PM: Male  CSP: Female  TP: Female  GC: Male  CAR: Female  CR: Female  CAE: Female  BHR: Male  CA: Female | 1 |
| 5. Experience and training | What experience or training did the researcher have? | GCM, KT, JS, RB, PM, TP, GC, CAR, CR, CAE, BHR are experienced researchers in qualitative studies and have collectively published numerous qualitative research articles. | 1 |
| *b*). *Relationship with participants* | | | |
| 6. Relationship established | Was a relationship established prior to study commencement? | Only for the purposes of this research. | N/A |
| 7. Participant knowledge of the interviewer | What did the participants know about the researcher? *e.g*. personal goals, reasons for doing the research | Participants did not know researchers personally but were aware about research purposes. | N/A |
| 8. Interviewer characteristics | What characteristics were reported about the interviewer/facilitator? *e.g*. Bias, assumptions, reasons and interests in the research topic | The characteristics of each author have not been reported. | N/A |
| Domain 2: study design | | |  |
| *a*). *Theoretical framework* | | | |
| 9. Methodological orientation and Theory | What methodological orientation was stated to underpin the study? *e.g*. grounded theory, discourse analysis etc | Focused ethnography and Thematic analysis | 8, 9 |
| *b*). *Participant selection* | | | |
| 11. Method of approach | How were participants approached? *e.g*. face-to-face, telephone, mail, email | Participant recruitment occurred through posters, in-person presentations by the research team, and snowball sampling. Family members of residents were recruited via invitations to LTC Family Councils. | 9 |
| 12. Sample size | How many participants were in the study? | 80 participants were recruited: 25 in interviews and 19 in focus groups, consisting of 20 healthcare aides, 14 licensed practical nurses (LPNs), 21 registered nurses (RNs), 10 LTC managers, 6 family members of residents, and 9 Emergency Medical Services (EMS) members. | 8 |
| 13. Non-participation | How many people refused to participate or dropped out? Reasons? | None | N/A |
| *c*). *Setting* | | | |
| 14. Setting of data collection | Where was the data collected? *e.g*. home, clinic, workplace | Data were collected at the Long-term care facilities. | 10 |
| 15. Presence of non-participants | Was anyone else present besides the participants and researchers? | No-one else was present besides the participants and the researchers. | N/A |
| 16. Description of sample | What are the important characteristics of the sample?  *e.g*. demographic data, date | Participants (healthcare aides, licensed practical nurses, registered nurses, LTC managers, family members of residents and Emergency Medical Services members) must have worked in the facility or service for at least one year, and had experience with one potentially avoidable transfer, or be a family member of a resident transferred to a local ED in the past year. | 8 |
| *d*). *Data collection* | | | |
| 17. Interview guide | Were questions, prompts, guides provided by the authors? Was it pilot tested? | The semi-structured interview/focus group guide was developed, but it was not pilot tested. | 9 |
| 18. Repeat interviews | Were repeat interviews carried out? If yes, how many? | No repeat interviews were carried out. | N/A |
| 19. Audio/visual recording | Did the research use audio or visual recording to collect the data? | Interviews were digitally recorded. | 9 |
| 20. Field notes | Were field notes made during and/or after the interview or focus group? | Field notes were taken during the interviews and focus groups. Interviewers also debriefed after the interviews and focus groups. | 9 |
| 21. Duration | What was the duration of the interviews or focus group? | The duration of the interviews ranged from 40 to 90 minutes, while the focus group discussions were approximately 1 to 2 hours. | 9 |
| 22. Data saturation | Was data saturation discussed? | When it appeared that we reached analytic saturation and no new information emerged during data collection^25^, we used the remaining two focus groups for verification of our findings. | 9 |
| 23. Transcripts returned | Were transcripts returned to participants for comment and/or correction? | The transcripts were not returned to participants for comment or correction. | N/A |
| Domain 3: analysis and findings | | |  |
| *a*). *Data analysis* | | | |
| 24. Number of data coders | How many data coders coded the data? | Three authors (RE, KT, JS) coded the data. | 9 |
| 25. Description of the coding tree | Did authors provide a description of the coding tree? | No | N/A |
| 26. Derivation of themes | Were themes identified in advance or derived from the data | The themes were derived from the data during analysis. | 9 |
| 27. Software | What software, if applicable, was used to manage the data? | NVivo 10 was used to manage the data. | 9 |
| 28. Participant checking | Did participants provide feedback on the findings? | No, participants did not provide any feedback on the findings. | N/A |
| *b*). *Reporting* | | | |
| 29. Quotations presented | Were participant quotations presented to illustrate the themes / findings?  Was each quotation identified? *e.g*. participant number | Yes, quotations were presented to illustrate theme. | 29-30 |
| 30. Data and findings consistent | Was there consistency between the data presented and the findings? | Yes, there was consistency between the data presented and the findings. | 11-13, 29-30 |
| 31. Clarity of major themes | Were major themes clearly presented in the findings? | Yes, major themes were clearly presented in the findings. | 11-13 |
| 32. Clarity of minor themes | Is there a description of diverse cases or discussion of minor themes? | No, there was no descriptions of the diverse cases or discussion of minor themes presented. | N/A |

Adapted from: Tong A, Sainsbury P, Craig J. Consolidated criteria for reporting qualitative research (COREQ): a 32-item checklist for interviews and focus groups. *International Journal for Quality in Health Care*. 2007. Volume 19, Number 6: pp. 349 – 357
